# Distributed computing for macromolecular crystallography

**DOI:** 10.1107/S2059798317014565

**Published:** 2018-02-01

**Authors:** Evgeny Krissinel, Ville Uski, Andrey Lebedev, Martyn Winn, Charles Ballard

**Affiliations:** a Scientific Computing Department, STFC, Rutherford Appleton Laboratory, Didcot OX11 0FA, England

**Keywords:** crystallographic computing, distributed computing, data and project management, web services, computational cloud

## Abstract

Modern crystallographic computing is characterized by the growing role of automated structure-solution pipelines, which represent complex expert systems utilizing a number of program components, decision makers and databases. They also require considerable computational resources and regular database maintenance, which is increasingly more difficult to provide at the level of individual desktop-based *CCP*4 setups. On the other hand, there is a significant growth in data processed in the field, which brings up the issue of centralized facilities for keeping both the data collected and structure-solution projects. The paradigm of distributed computing and data management offers a convenient approach to tackling these problems, which has become more attractive in recent years owing to the popularity of mobile devices such as tablets and ultra-portable laptops. In this article, an overview is given of developments by CCP4 aimed at bringing distributed crystallographic computations to a wide crystallographic community.

## Abstract 2

The paper describes recent CCP4 initiatives and projects aimed at bringing software and data services which utilize distributed computational resources to users.

## 1. Introduction   

During the last decade, there has been remarkable progress in methods for structure solution, in large part associated with the development of automated expert systems that are capable of delivering almost complete solutions with minimal or no user intervention. Such systems represent complex scripts (commonly called pipelines) built on top of more elementary programs. Such automation is based on three fundamental components: intrinsic knowledge (expressed in the algorithms and decision makers that are used); extrinsic knowledge (coming from external sources such as structure and sequence databases); and sufficiently deep exploration of known approaches and various parameters.

All robust structure-solution pipelines are intrinsically complex and, algorithmically, aggregate expertise accumulated from many use cases. They can perform rather complex multi-stage actions, which once required a sufficiently high level of user expertise. One of the first essentially automated pipelines, *Auto-Rickshaw* (Panjikar *et al.*, 2005 ▸), was initially created in order to provide a quick answer to the question of whether a diffraction experiment was successful or not, but it then became a rather comprehensive and complex tool available through a dedicated webserver. *Auto-Rickshaw* starts with data processing and calculates a density map using experimental phasing techniques (SAD, MAD or SIRAS). The resulting density map could be interpreted using automated model building with the *ARP*/*wARP* server (Langer *et al.*, 2008 ▸) or *Buccaneer* (Cowtan, 2006 ▸). Another example of a crystallographic server is given by *PDB_REDO* (Joosten *et al.*, 2014 ▸) for the final refinement and validation of solved structures.

*CCP*4 includes several tools for automated structure solution. The molecular-replacement (MR) pipeline *BALBES* (Long *et al.*, 2008 ▸) consolidates expert knowledge in both phasing and model preparation. For example, it may construct multimeric molecular models if there is an indication of possible protein oligomerization and doing so increases the chances of solution. Finding suitable structural homologues is crucial for the success of MR, and therefore MR pipelines come with databases of conveniently prepared structural domains [*BALBES* and *MoRDa* (Vagin & Lebedev, 2015 ▸)] or try to construct models from raw PDB (Protein Data Bank; Berman *et al.*, 2000 ▸) files (*MrBUMP*; Keegan & Winn, 2008 ▸). When a suitable structural homologue cannot be found, *AMPLE* (Bibby *et al.*, 2012 ▸) uses the *ROSETTA* (Rohl *et al.*, 2004 ▸) or *QUARK* (Xu & Zhang, 2012 ▸) structure-modelling software to build molecular models from the sequence. This procedure is based on sizeable databases of structural fragments included in the *QUARK* and *ROSETTA* setups. Yet, the main power of automatic solvers remains in the exploration of different approaches and variation of intrinsic parameters, which results in relatively long calculation times.

Table 1 ▸ presents the principal automatic structure-solution pipelines distributed by *CCP*4. As can be seen from the table, most of them require considerable CPU resources and the combined size of the databases required is close to 40 GB. In addition, *AMPLE* requires third-party software (*ROSETTA* and *QUARK*), which needs to be installed and maintained separately. While these requirements are suitable for medium-size laboratories, individual researchers and small groups may find the setup and associated maintenance burdensome.

The specifications of crystallographic structure-solution pipelines, as outlined in Table 1 ▸, suggest that they are more suited to a server-type deployment as against traditional local setups on personal PCs. Server-based setups have obvious advantages in that they eliminate the setup and maintenance burden for users while providing access to CPU and data resources. Yet, there are other factors which may have an influence on the suitability of remote computations for end users. Apart from restrictions on transmitting data to remote servers, which are commonly met in both academic and industrial environments, one of the greatest inconveniences may be seen in changing between different (remote and local) working environments. Unless implemented properly, such switchovers leave users fully responsible for all associated data management and keeping their projects in a consistent state suitable for possible examination at a later date.

In this paper, we report the various approaches being developed by CCP4 to providing distributed crystallographic computation environments. The first approach is based on a traditional web-server paradigm, in which particular pipelines are framed as web services, communicating with the user through file upload and download. The second approach employs conventional *CCP*4 setups in a computational cloud, with the facility to submit CPU-heavy jobs for remote computation from the *CCP*4 *Graphical User Interface* 2 (*CCP*4*i*2; Potterton *et al.*, 2018 ▸). The third line is represented by a specialized web application, which may be seen as a combination approach, having elements of both traditional web services and cloud setups. In the following, we describe the details and functionality of each line of development and provide their comparative analysis from both user and maintenance points of view.

## 2. *CCP*4 web services   

**Figure 1:**
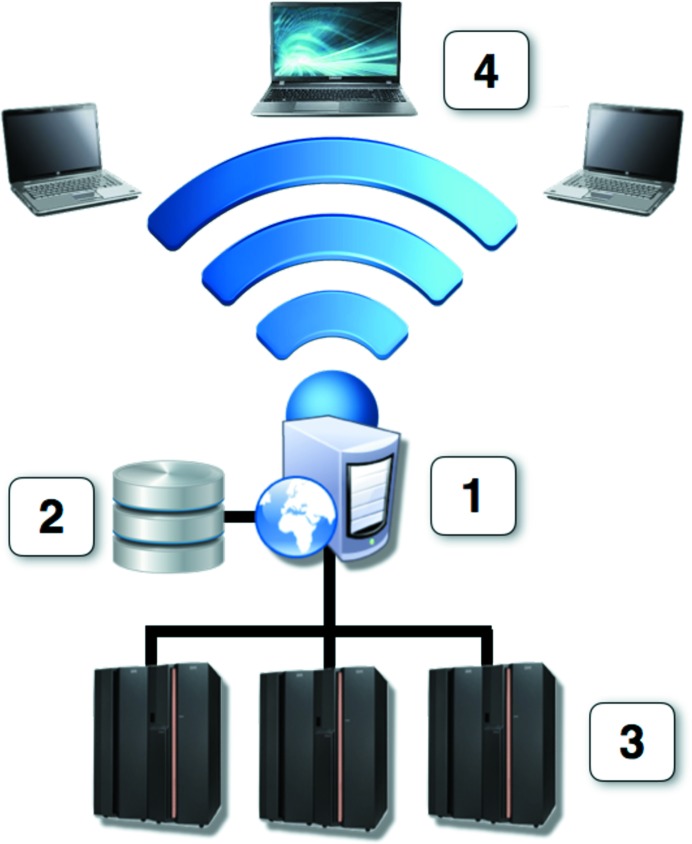
Schematic of a conventional web-service setup. The setup contains four basic elements: (1) a web-server machine, (2) data storage, (3) computational machines connected to the web server *via* an internal network and (4) client devices communicating with the web server *via* http or https protocols. (1), (2) and (3) can all be placed on physical machines individually or shared in any combination.

Automatic pipelines for structure solution, which consume significant CPU resources and utilize custom databases, are naturally suitable for deployment as web services. Fig. 1 ▸ presents a schematic of a conventional web-service setup, which is characterized by the presence of a single internet portal (1, the web server), data storage for keeping user data and calculation results (2, the database), and number crunchers (3). Depending on the anticipated workload, elements of the web service may be deployed on individual machines, or combined on fewer machines to optimize hardware resources. It is not uncommon to keep the database and web server on the same machine, although this is not desirable for security reasons.

Since web services act as a shared resource, they typically maintain user accounts with or without quotas for disk storage and CPU time. In contrast to web applications, interactivity is not an assumed property of web services, although certain interactive elements, such as molecular graphics (*CCP*4*mg*; McNicholas *et al.*, 2011 ▸), can be used as part of the report system. In the most straightforward implementation, a web service is expected to provide a user with facilities to upload data, start calculations and return results as a web page or/and downloadable file or bundle of files.

**Figure 2:**
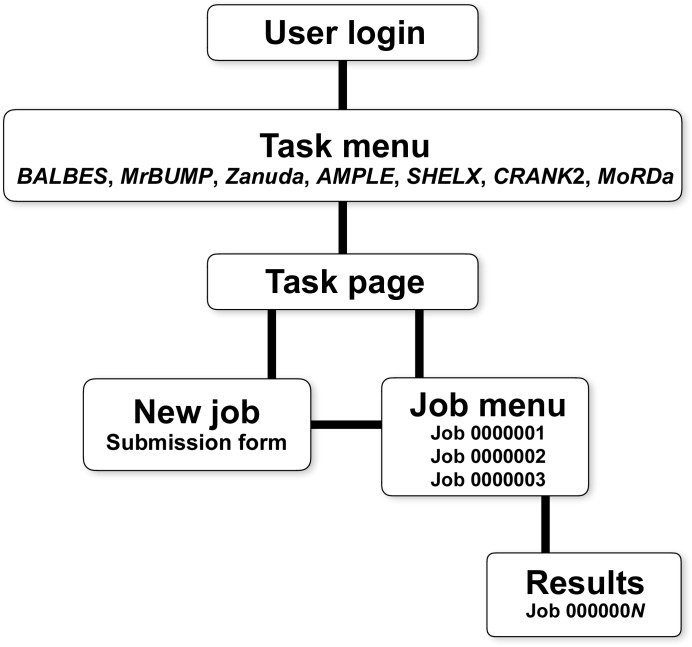
General workflow of *CCP*4 web services.

The first *CCP*4 web service, *BALBES*, was released in 2008 (Long *et al.*, 2008 ▸) with a setup closely matching that in Fig. 1 ▸ and with general specifications as described above. Further web services were then gradually added following designer solutions largely borrowed from the first *BALBES* implementation. The overall workflow of *CCP*4 web services is shown in Fig. 2 ▸. In order to use the services, a user must be registered with the system, which is necessary in order to ensure that users can see only their own results and access only their own data. After login, a user chooses one of the available web services (tasks) to work with. For each task, the corresponding page contains a list of finished and running jobs, as well as a link to the submission form for a new job. The list of jobs contains links to individual pages with job results, where a user can see the job summary and log files, and can download the resulting MTZ and/or PDB files. Where a web service produces a considerable number of intermediate or alternative results (for example multiple molecular models in automatic MR pipelines), they are presented through a navigation tree replicating the structure of the actual working directory.

At present, *CCP*4 web services contain eight applications: three automatic molecular-replacement pipelines (*BALBES*, *MrBUMP* and *MoRDa*), two experimental phasing pipelines [*CRANK*2 (Skubák & Pannu, 2013 ▸) and *SHELX* (Sheldrick, 2015 ▸)], MR with *ab initio* modelling (*AMPLE*), a space-group validation pipeline (*Zanuda*; Lebedev & Isupov, 2014 ▸) and crystal-packing and oligomerization analysis (*jsPISA*; Krissinel, 2015 ▸). In routine cases (no crystal pathologies, complete and good resolution data, correct sequence and a highly similar structural homologue), MR pipelines will find correct orientations and positions of molecules in the asymmetric unit and a good approximation to the phases. *BALBES* and *MoRDa* use conceptually similar databases of single-domain MR models derived from the PDB in a semi-automatic way. In the case of multi-domain and complex targets, they will also automatically construct multimeric models and use data generated by *PISA* (Krissinel & Henrick, 2007 ▸) for complexes. In contrast to these pipelines, *MrBUMP* creates MR models from the current PDB archive, which eliminates the need for database support but may result in longer calculation times. After phasing, the coordinates and density maps obtained can be sent automatically to the *ARP*/*wARP* server at EMBL Hamburg for model rebuilding and refinement. Successfully built models can be sent further to the *PDB_REDO* server for completion (final refinement and validation). Alternatively, the structure may be downloaded and rebuilt/corrected at any stage using the *Buccaneer*, *Coot* (Emsley & Cowtan, 2004 ▸) and *REFMAC* (Murshudov *et al.*, 2011 ▸) software from *CCP*4.

A significant advantage of the *CCP*4 online web services is that they are very simple to use. Starting a job requires the completion of a simple submission form to upload merged reflection data and sequences (or a coordinate file in the case of *Zanuda* and *jsPISA*) followed by pushing a ‘submit’ button. Technically, *CCP*4 web services are relatively simple as well and require no special software to be installed. In addition, the services work well with modest broadband speeds (100–200 kB s^−1^) and require minimal maintenance effort. On the other hand, they represent only a limited slice of *CCP*4 functionality and, in contrast to the desktop GUI, do not support structure-solution projects and have no data framework. They can be used only to obtain a first approximation to the target structure suitable for further structure completion and ligand fitting with the ordinary (offline) *CCP*4 setup. Currently, *CCP*4 web services are powered by an in-house 196-core cluster and complete about 500 structure-solution queries every month.

## 3. *CCP*4 Cloud   

The computational cloud (Hassan, 2011 ▸) represents a new model for everyday computing which is becoming increasingly popular. Recent years have been marked by an explosive development and spreading of ultraportable devices, such as tablets, Chromebooks and smartphones, which may be seen as mere cloud terminals. There is also a clear trend towards cloud-based operations in software installed on conventional desktops and laptops. Although cloud computing may be seen as less suitable in terms of data security, for the majority of users it offers at least four essential advantages: (i) the elimination of local data maintenance; (ii) the elimination of data exchange between user devices; (iii) independence of the type of user device; and (iv) access to considerable computational resources.

A pure cloud model assumes full hardware virtualization (Smith & Nair, 2005 ▸) with remote access [usually *via* http(s) protocol] to the respective virtual machines [VMs; in practice, more lightweight software containers, such as *Docker* (Mouat, 2015 ▸), may be used instead of a full VM]. Such VMs are created from prepared images that have all necessary software pre-installed and usually exist during the user session only. When a job needs to be submitted, a dedicated number-crunching VM or a virtual cluster is created. Large-scale clouds, such as Amazon or Azure, operate rather closely to these principles.

**Figure 3:**
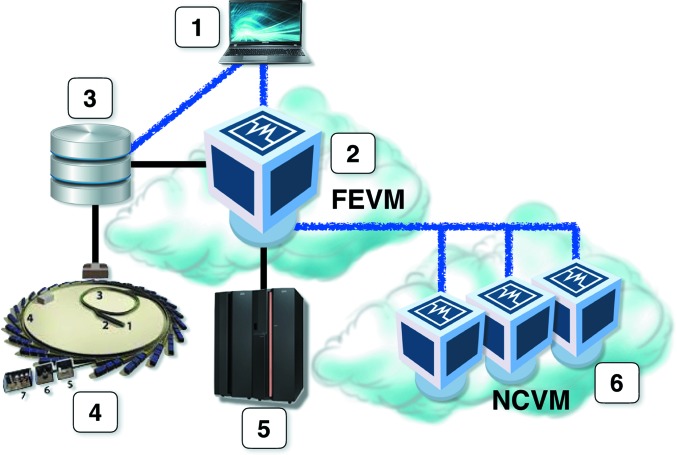
*CCP*4 Cloud schematic. (1) Client device. (2) Front-end virtual machine. (3) Persistent data storage. (4) Data-producing facility (for example a synchrotron). (5) Local number-crunching facility. (6) Number-crunching virtual machines. Black lines indicate in-house communications; blue fuzzy lines correspond to external http(s) connections.

**Figure 4:**
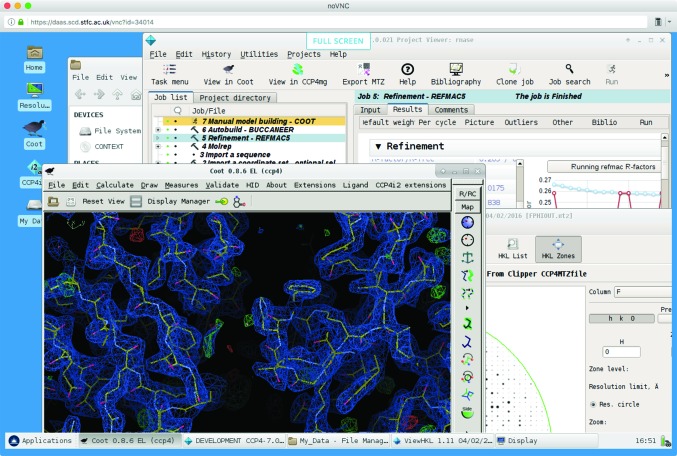
A snapshot of the *CCP*4 Cloud FEVM desktop.

It appears that a ‘pure cloud’ model is not the most convenient one for crystallographic computations with the *CCP*4 software suite. While a VM image with *CCP*4 set up can be created, one needs to associate a VM instance with persistent storage in order to keep all user data and projects between sessions, and to receive results from number crunchers even if the user session is closed. Fig. 3 ▸ presents a schematic of the *CCP*4 Cloud setup, which is currently being constructed by CCP4 in association with the Scientific Computing Department of the Science and Technologies Facilities Council UK (STFC/SCD). As shown in the figure, the setup is a mixture of virtualized and ‘real’ elements. A user (1) connects to a front-end virtual machine (FEVM) (2), which presents the user with a Linux desktop (Fig. 4 ▸). The virtual machine is created from a specially prepared image, which contains the Linux OS and pre-installed *CCP*4 suite, and also a mount link to the user’s own storage area (3). The storage area exists independently of the front-end machine, and is also accessible through a shared folder from the user’s own device, such that all necessary data [merged and unmerged MTZ files, coordinate files (PDB or mmCIF), sequence files, ligand descriptors and others] can be uploaded. Uploaded files appear in the front-end VM’s file system and may be used *via* *CCP*4*i*2 in exactly the same way as in a local *CCP*4 setup. X-ray diffraction images could also be uploaded to the user’s storage, but this is less convenient because of the high data volumes involved. In the future, images will be loaded directly into the user’s persistent storage from data-producing facilities, such as a synchrotron beamline (4), using a dedicated transfer protocol. STFC/SCD keeps an archive of all data collected at Diamond Light Source, which will be made available to the *CCP*4 Cloud using the iCAT (Flannery *et al.*, 2009 ▸) data catalogue. The corresponding developments are in progress.

The front-end VM can run *CCP*4 jobs in the same manner as an ordinary desktop. This is probably the best option for jobs taking no more than 5–10 min, such as molecular-model preparations; quick molecular replacement with *MOLREP* (Vagin & Teplyakov, 2010 ▸) or *Phaser* (McCoy *et al.*, 2007 ▸); a few cycles of refinement with *REFMAC*; density modifications with *Parrot* (Cowtan, 2010 ▸); *PISA* analysis; *GESAMT* structural alignments and database searches (Krissinel, 2012 ▸); and similar. Manual model building with *Coot* is also performed directly in the FEVM. Longer jobs, however, such as automatic MR solvers (*MrBUMP*, *BALBES*, *MoRDa*, *AMPLE* and *Phaser* in expert mode), EP pipelines (*CRANK*2 and *SHELX*), automatic model building with *Buccaneer* and some others, need additional computing resources. At present, *CCP*4 Cloud uses the SCARF facility at STFC/SCD, which is a multi-core computational cluster. In order to run a job on SCARF, the user should activate the job using the special ‘Run on server’ button found in the *CCP*4 GUI toolbar. When a job is dispatched to the remote computing facility, all of the job’s input data are packed in a jobball together with the running instructions and sent to the head node of the cluster, from where it is submitted to the queueing system. After calculations are finished, all results with the corresponding metadata are again packed in a ‘jobball’, which is then retrieved by FEVM, unpacked and checked in the *CCP*4*i*2 database, after which the results may be viewed by the user.

Although the current implementation of *CCP*4 Cloud is based on using SCD’s SCARF facility for computationally expensive jobs, it does not preclude the inclusion of other cloud resources, such as the Amazon and Azure clouds, and the European Grid Initiative (EGI; Kranzlmüller *et al.*, 2010 ▸), in the setup [facility (6) in Fig. 3 ▸]. In this case, the number-cruncher VM should be created, upon job instantiation, from a prepared image containing the full *CCP*4 setup. The jobball-based communication protocol described above can then be extended for using such resources, and the corresponding developments are currently under way.

The *CCP*4 Cloud scheme has a significant advantage in that it delivers full *CCP*4 functionality and is operated *via* the same *CCP*4*i*2 interface that users are familiar with. *CCP*4 Cloud projects are fully compatible with those a user may have on local machines, which may be inter-exchanged with the Cloud *via* *CCP*4*i*2’s dedicated export/import facilities. At the same time, *CCP*4 Cloud requires substantial computational resources for running FEVMs and a fast and stable internet connection, and relies on the availability of IT expertise for setting up virtual machines, their configuration and everyday maintenance. At present, there is no possibility of providing a ready out-of-box configuration of *CCP*4 Cloud, as many details of each setup will depend on the technical features of a particular cloud implementation. Although versatile and convenient for users, *CCP*4 Cloud may only be used at large-size facilities with an appropriate level of hardware infrastructure and IT support.

## 4. *CCP*4 web application   

Web application is a generic term for programs served from remote locations but with front ends running on client machines through a web browser (Shklar, 2009 ▸). Typically, the front end and part of the server code of such applications are written in Java or Javascript, and client–server communication is based on AJAX (Asynchronous Javascript and XML) technologies. Today, web applications successfully compete with ordinary desktop applications and provide an equivalent GUI experience, owing to the ability of modern web browsers to create rich graphical content programmatically.

**Figure 5:**
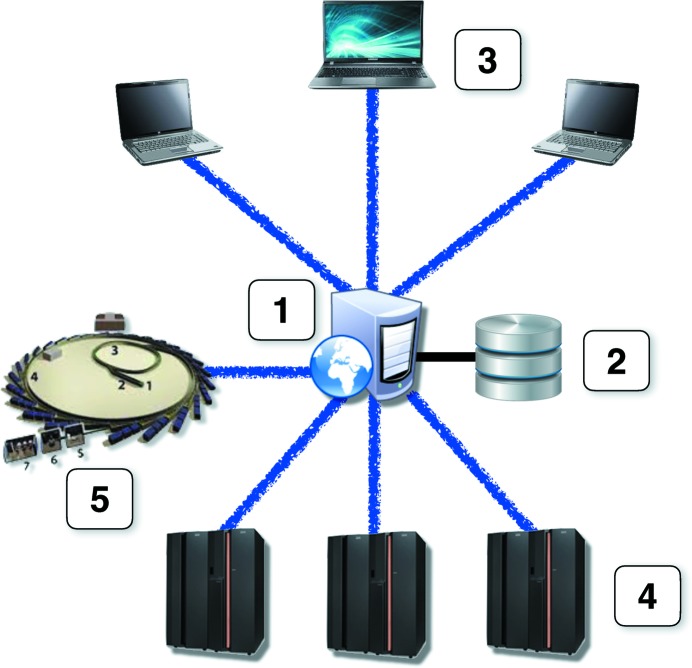
Schematic of the *CCP*4 web application. (1) Front-end machine (FE). (2) Data storage. (3) Client machines with optional local servers. (4) Number-cruncher servers (NCs). (5) Data-producing facility. Black lines indicate in-house communications; blue fuzzy lines correspond to http(s) connections.

Over the last decade, a number of frameworks have been created in order to facilitate the development of professional-looking web applications [jQuery (Duckett, 2014 ▸), Dojo (Russell, 2008 ▸), GWT (Tacy *et al.*, 2013 ▸), Angular (Seshadri & Green, 2014 ▸) and many others], which make the task no more complex than creating ordinary desktop GUIs with Qt (Lazar & Penea, 2016 ▸), Wx (Smart *et al.*, 2005 ▸), GTK+ (Krause, 2007 ▸) or similar. However, web applications may have a number of architectural solutions depending on the nature of the tasks solved, the anticipated number of users working simultaneously, CPU and storage demands and desired scalability. For the *CCP*4 web application *jsCoFE* (JavaScript-powered Cloud Front End), we have chosen the multi-server architecture presented in Fig. 5 ▸. At first glance, it is rather similar to the ordinary web-service setup presented in Fig. 1 ▸, and may be seen as a version in which in-house communications between the web server and number crunchers (NCs) are replaced with http(s) connections. The latter makes the system extremely scalable, as it allows NCs to be placed anywhere and plugged in by a mere adjustment of configuration files on the front-end (FE) machine.

The FE machine should be based on a reasonably powerful server, which works as a hub for all communications in the system. All work on forming job packages and maintaining user projects is performed on the corresponding client machines, which exchange only brief instructions and metadata (apart from uploading all necessary data such as MTZ and PDB files and sequences) with the FE, usually at a few kilobytes every few seconds. This type of communication is very light and does not affect the interactivity of the client GUI even at low broadband speeds (50–100 kB s^−1^). Communication between the FE and NCs involves jobballs containing each job’s data and metadata (usually 10–30 MB) and therefore should be sufficiently fast. In practice, using an internal 1 Gb network causes no obvious delays, especially in comparison with the usual lags associated with starting a job on computational clusters. Communicating with external NCs *via* modern 50–100 Mb broadband connections could result in few-second to minute transmission times, which are still perfectly acceptable for CPU-intensive jobs that take from a few hours to days or weeks to run. Communication with the data-producing facility (5) is necessary mostly for acquiring diffraction images directly, in order to avoid the upload of image data of a few gigabytes in size from client machines. This part of *jsCoFE* is currently under development.

A *jsCoFE* setup should be able to serve from tens to hundreds of simultaneously working users without loss of interactivity on client machines. This cannot be achieved with the CGI-based techniques (Boutell, 1996 ▸) commonly used in traditional web services, which launch a separate process in answer to each request coming to the server. Instead, the server application should always be active in order to eliminate the startup time. It is expected that most communications will involve a measurable transmission time but a negligible number of CPU cycles. Therefore, all communications should be asynchronous, leaving the FE free and responsive while data are being transmitted. These requirements are fulfilled by Node JS (Cantelon *et al.*, 2013 ▸), which was chosen as a platform for all server-side developments. The client part of the system, *jsCoFE*, is written in Javascript using the jQuery framework. *jsCoFE* supports user accounting, authentication and login. Each user account contains a list of projects, which is shown immediately after login. Each project is presented as a tree hierarchy of jobs based on the parent–child relations of the jobs automatically derived from data flows.

**Figure 6:**
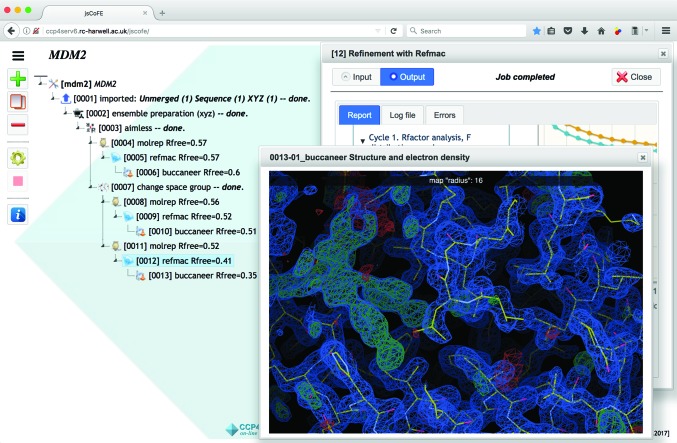
A snapshot of the *jsCoFE* project window.

Fig. 6 ▸ presents a snapshot of an example *jsCoFE* project, giving an idea of its basics. All jobs in the project tree are selectable and right/left-clickable. The toolbar on the left-hand side of the tree allows the currently selected job to be added, cloned, deleted, opened and stopped, and access to documentation. The toolbar is also available as a drop-down menu by right-clicking on a job line. Opening a job (by double-clicking on the job line) displays the Job Dialog, which contains the Input and Output tabs. On input, the job will see all data imported or produced up the tree branch, which can be selected with the corresponding drop-down lists. If there are insufficient data of the correct type for the job (for example no molecular-replacement models were imported or generated), then the job will not be created and an explanatory message is shown instead. The output page can display the structured report, containing graphs, tables and sections; all structural data and density maps can be displayed using the *UglyMol* software (Wojdyr, unpublished work). Report pages may be created programmatically as part of Python wrappers written to execute tasks on NCs, or generated automatically using *CCP*4 log-file markup (‘Baubles markup’). Full log files are displayed in a separate output tab for detailed inspection, if necessary. *jsCoFE* may optionally include local servers, running on client machines, for launching locally installed *CCP*4 desktop applications such as *Coot*, *CCP*4*mg* or *ViewHKL* (Evans & Krissinel, unpublished work); the corresponding buttons appear automatically in report pages if a local server is detected. When a local server is present, *jsCoFE* also allows model-building tasks using *Coot* from the *CCP*4 suite installed on the client. *jsCoFE* is currently under active development; Table 2 ▸ lists the tasks available in the development version.

The *CCP*4 web application should be seen as a deep modernization of the *CCP*4 web services, and will probably replace them at some point in the future. It is superior to plain web services in that it supports projects as a hierarchy of related jobs leading to structure solution, and it also presents the user with a richer interface and a more complete list of tasks. On the other hand, it is somewhat more complex as an application, and if a user wishes just to run *BALBES*, for example, then the corresponding web server allows such a job to be started in fewer clicks. Technically, *jsCoFE* is a highly portable application, which can be installed locally with minimal or no help from IT support. Adding new tasks to *jsCoFE* is based on the developed framework and, in principle, can be performed by an experienced programmer familiar with development in Javascript and Python. Using the jQuery library for client-code development makes it suitable for use on both desktops/laptops and mobile devices.

## 5. Discussion   

Distributed computations become increasingly more attractive an alternative to the more traditional approach based on the possession and maintenance of local resources. Associated benefits include not only access to potentially unlimited computational power, but also to a centrally maintained *CCP*4 setup, databases and user projects available on any device without the need for explicit export and import. The only drawbacks of a distributed model include the requirement for permanent internet access and concerns regarding data security. While the former is a next-to-negligible factor these days, the latter may still be a factor of deterrence in corporate environments. However, even there distributed computing may be employed locally, for example within the network of a particular company or a university laboratory.

Realising many benefits of distributed computing for end users, CCP4 is exploring a few approaches, described above, which may potentially appeal to the crystallographic community. Table 3 ▸ summarizes the key factors which we used to estimate their practicality from the points of view of both users and maintainers/developers. The resulting picture is rather mixed, and it is not possible to unambiguously identify the most suitable approach. The weakest point of traditional web services appears to be the absence of user projects; however, this makes the user interface much easier, where any task can be run in just a few clicks, which may be seen as advantage by some users. Adding user projects to web services would make them equivalent to the *CCP*4 web application, which currently loses to *CCP*4 Cloud in terms of functionality; in particular, the interactive model-building tool (*Coot*) is missing there. *CCP*4 Cloud is the most convenient solution from the narrow perspective of *CCP*4 core maintainers, because it levies all the complexity of setup and maintenance on local IT support. However, running the *CCP*4 desktop GUI in a browser window is not the most convenient option for mobile device users owing to the specifics of desktop graphical widgets and the extensive use of left/right mouse clicks. Cloud setups are also demanding in terms of hardware and bandwidth, which limits their deployment to sites with a high level of computing infrastructure.

## 6. Current availability   

Most of the work presented in this paper is currently in progress and the corresponding software and service will be released in the shortest time subject to progress. *CCP*4 web services are fully available at https://www.ccp4.ac.uk/ccp4online/ with free registration. *CCP*4 Cloud is in test mode; registration is available on demand to users with a UK FedID. The *CCP*4 web application, *jsCoFE* (http://rcccp4serv1.rc-harwell.ac.uk/jscofe/), is being prepared for alpha release and is available for test volunteers upon registration at the *jsCoFE* home page.

## 7. Conclusion   

In the present publication, we have given an overview of the current CCP4 activities towards distributed computations for the MX crystallographic community. While ordinary desktops and laptops will be in use for the foreseeable future, the rising popularity of mobile devices and cloud-based approaches to data and software services is fairly obvious. With respect to structure-solution tasks requiring access to supercomputing and databases, the move to cloud models is already recognized as a necessity. From the approaches that we have discussed, *CCP*4 Cloud represents a good solution for corporate setups with a developed computing infrastructure. *CCP*4 web application is a relatively lightweight solution that is easy to install and maintain virtually anywhere, but it needs further development to match the functionality of *CCP*4 Cloud. *CCP*4 web services remain a well targeted collection of useful CPU-intensive tasks with easy and efficient access, obviously appealing to its ∼600 regular users. It may take a few more years before clarity is reached on which approach, if any, will appeal to *CCP*4 users most.

## Figures and Tables

**Table 1:** The main automated crystallographic pipelines distributed by *CCP*4

Pipeline	Description	Use of databases	Expected CPU time
*xia*2	Data processing	0	Minutes to hours
*BALBES*	Molecular replacement	3 GB	Hours to days
*MoRDa*	Molecular replacement	3 GB	Hours to days
*MrBUMP*	Molecular replacement	26 GB	Hours to days
*AMPLE*	*Ab initio* modelling and molecular replacement	*ROSETTA*	Days to weeks
*SIMBAD*	Contaminant searches and sequence-less MR	25 GB	Hours to days
*CRANK*2	Experimental phasing (SAD, SIRAS, MAD)	0	Hours to days
*ARCIMBOLDO*	Fragment-based MR	∼1 GB	Hours to weeks
*Phaser*	Automated phasing	0	Minutes to weeks
*Buccaneer*	Model building	0	Minutes to hours
*Nautilus*	DNA/RNA model building	0	Minutes to hours

**Table 2:** *CCP*4 tasks currently available through *jsCoFE*

Task	Description
Data import	Import of project data: merged and unmerged MTZ files, PDB/mmCIF files, sequence files. The file type is recognized automatically and all data are arranged in data sets at the metadata level, such that all subsequent jobs can operate with data sets rather than explicit reference to files. For example, MTZ files can be logically split into several data sets, PDB files may be split into chains, and files with multiple sequences are split into individual sequence entities.
Convert to structure	Association of coordinate and density/phase data, usually after data import. In *jsCoFE*, coordinates and density maps are associated to optimize data flows.
POINTLESS/AIMLESS	*POINTLESS*/*AIMLESS* pipeline for scaling and merging data
Reindex	Changing the space group, usually required in the case of reflection-data enantiomorphism
AsuDef	Definition of the asymmetric unit and Matthews analysis
BALBES	Automatic molecular replacement with *BALBES*
MoRDa	Automatic molecular replacement with *MoRDa*
MR ensembling from sequence	Making MR model ensembles through sequence searches in the PDB
MR ensembling from coordinates	Making MR model ensembles from given coordinate data
MOLREP	Molecular replacement with *MOLREP*
Phaser-MR	Molecular replacement with *Phaser*
SHELX-MR	After-MR autotracing with *SHELXE*
CRANK2	Automatic experimental phasing with *CRANK*2 (SAD, MAD, SIRAS)
SHELX-auto	Automatic experimental phasing (SAD, MAD, SIRAS) using the *SHELX* suite
SHELX-Substr	Heavy-atom location with *SHELXC*/*D*
Phaser-EP	Experimental phasing with *Phaser*
Parrot	Density modification with *Parrot*
REFMAC	Macromolecular refinement with *REFMAC*
LORESTR	Low-resolution refinement pipeline
Buccaneer-MR	Automatic model building with *Buccaneer* after MR
MakeLigand	Making ligand structure and restraints with *AceDRG*
FitLigand	Fitting ligand with *Coot*
FitWaters	Fitting water molecules with *Coot*
Zanuda	Space-group validation with *Zanuda*
GESAMT	Pairwise and multiple structural alignment and structural searches in the PDB with *GESAMT*
PISA	Oligomeric state and interface analysis with *PISA*

**Table 3:** Comparison of the discussed approaches to distributed computing models and the traditional *CCP*4 desktop setup in crystallography

Criteria	Web services	*CCP*4 Cloud	*CCP*4 web application	Traditional desktop setup
Functionality	Limited to automatic structure-solution pipelines	Full	Subject to the level of development; currently lacks a number of tasks and interactive graphical tools (*Coot*)	Full
Interface complexity	Easy to use	Usual	Usual	Usual
User projects support	No	Yes	Yes	Yes
Bandwidth requirements	Low	High	Medium to low	N/A
Suitability for standalone use on desktops	Not suitable	Can execute jobs on remote servers from ordinary *CCP*4 setups	Allows desktop setups in flexible configurations	Fully suitable
Suitability for use on mobile devices	Yes	Not very suitable owing to the specific graphical design and extensive use of mouse	Yes	No
Hardware requirements	Low beyond number-crunching facilities	High in addition to number-crunching facilities	Low beyond number-crunching facilities	Medium for most tasks; high for automatic structure solvers
Additional software requirements	None	High	Low	N/A
Portability (suitability for corporate deployment)	May require a custom installation	Requires IT support with cloud setup experience	High	N/A
Maintenance burden	Low	Dependent on local IT support	Low	Low to medium
